# Expansion of invariant natural killer T cells from systemic lupus erythematosus patients by alpha-Galactosylceramide and IL-15

**DOI:** 10.1371/journal.pone.0261727

**Published:** 2021-12-22

**Authors:** Chien-Ya Hsu, Yu-Shan Chueh, Ming-Ling Kuo, Pei-Tzu Lee, Hsiu-Shan Hsiao, Jing-Long Huang, Syh-Jae Lin

**Affiliations:** 1 Division of Asthma, Allergy, and Rheumatology, Department of Pediatrics, Chang Gung Memorial Hospital, College of Medicine, Chang Gung University, Taoyuan, Taiwan; 2 Department of Microbiology and Immunology, Graduate Institute of Biomedical Sciences, College of Medicine, Chang Gung University, Taoyuan, Taiwan; 3 Center for Medical and Clinical Immunology, College of Medicine, Chang Gung University, Taoyuan, Taiwan; 4 Department of Pediatrics, New Taipei Municipal Tu Cheng Hospital, Chang Gung Memorial Hospital and Chang Gung University, Taoyuan, Taiwan; Cornell University Joan and Sanford I Weill Medical College, UNITED STATES

## Abstract

CD1d-restricted invariant natural killer T cells (iNKT cells) may play an important role in the pathogenesis of systemic lupus erythematosus (SLE). Interleukin (IL)-15 is a pro-inflammatory cytokine which is over-expressed in SLE patients. In the present study, we investigated the iNKT cell expansion of mononuclear cells (MNCs) from SLE patients following 10 days’ culture with α-galactosylceramide (α-Galcer) and /or IL-15. We sought to determine the phenotypic and functional characteristics of the expanded iNKT cells compared to healthy controls and correlated with disease activity. We observed that 1. The percentages of Vα24+/Vβ11+ iNKT cells following 10-day incubation was lower in SLE groups compared to controls; 2. The percentages and absolute numbers of Vα24+/Vβ11+ iNKT cells were expanded by α-galactosylceramide (α-Galcer), and further enhanced with IL-15 in SLE patient, but the effect of IL-15 was much lower than controls; 3.IL-15 +α-Galcer expanded CD3+/CD56+ NKT-like cells from SLE patients, especially with active disease 4. The CD161+ Vα24+/Vβ11+ iNKT cells in SLE were more responsive to α-Galcer stimulation than the CD161- counterpart; 5. IL-15 decreased apoptosis of α-Galcer activated SLE iNKT cells; 6. IL-15 enhanced CD69, CD1d and CD11a expression on α-Galcer treated iNKT cells; 7. The IL-4 production of iNKT cells was decreased in SLE patients compared to controls; 8. IL-15 increased IFN-γ and IL-4 production of SLE iNKT cells; 8. IL-15 failed to augment the ability of iNKT cells to aid NK-mediated K562 cytolysis in SLE patients; 9. CD161 positivity, granzyme B and perforin expression of α-Galcer+IL-15 expanded iNKT cells correlated with C3 levels in SLE patients. Taken together, our results demonstrated numeric and functional deficiency of iNKT cells and their response to IL-15 in SLE patients. Our finding may provide insight for using adoptive iNKT cell therapy in autoimmune diseases.

## Introduction

Invariant natural killer T (iNKT) cells are a group of innate non-conventional T cells which recognized glycolipids presented by CD1d. They express a unique invariant TCRα chain (Vα24Jα18 in humans) and are activated by the glycolipid α-galactosylceramide (α-Galcer) [1, 2]. Peripheral iNKT cells comprise less than 1% of peripheral blood T cells and share similar functional and phenotypic features with natural killer (NK) cells. Upon CD1d-restricted activation, iNKT cells promptly secrete large quantities of interferon-γ (IFN-γ) and interleukin-4 (IL-4) that subsequently activate T-, B-, and NK-cells [3, 4]. Therefore, iNKT cells play a pivotal role in immune regulation, providing effective anti-microbial and anti-tumor defense.

Systemic lupus erythematous (SLE) is a systemic autoimmune disease. Autoantibodies against self-antigen can be detected in SLE patients which implicate the dysfunction of B cell immune response [5]. The number of invariant TCR Vα24Jα18^+^ T cells are reduced in peripheral blood lymphocytes of patients with SLE [6, 7]. Poor proliferative response of iNKT cells to α-GalCer was found in SLE patients compared to controls [8]. iNKT cell deficiency correlates with SLEDAI (a marker of disease activity) [9], suggesting that iNKT cells are important involved in the control of disease.

Interleukin (IL)-15, a γ-chain signaling cytokine essential for NK cells survival, serves as an immune regulator for both innate and adaptive immune response [10]. IL-15 also regulate homeostasis and terminal maturation of iNKT cells [11, 12]. IL-15 combination with IL-12, compensated for the selective loss of iNKT cells in HIV infection [13]. We have shown in our previous studies that IL-15 synergized with α-Galcer in expanding iNKT cells from umbilical cord blood MNCs [14]. We also found that the serum IL-15 levels were high in SLE patients and correlated with disease activities [15].

In the present study, we determine the efficacy of IL-15, in combination with α-Galcer to expand iNKT cells and enhance their function in SLE patients. We also sought to examine the numeric, phenotypic and functional characteristics of iNKT cells in SLE patients compared to normal controls.

## Material and methods

### Study subjects

This study protocols were reviewed and approved by the institutional review board of the institutional of review board of Chang Gung Memory Hospital (Institutional of Review Board No. 201601816A3). Study subjects include 26 SLE patients and 13 healthy controls recruited from Chang Gung Memorial Hospital (CGMH), Linkou, Taiwan (Table 1). The diagnosis of SLE fulfills the 1997 American College of Rheumatology classification criteria [16]. We evaluated the severity of our SLE patients using the systemic lupus erythematosus disease activity index (SLEDAI) scoring method [17], a score greater or equal to 6 defined active disease. The demographic characteristics of SLE patients are shown in Table 1, including sex, age, SLEDAI score, C3, C4, anti-dsDNA, and percentages of lupus nephritis and the number of circulating iNKT cells. We obtained heparinized whole blood for retrieval of mononuclear cells (MNCs) from each individual under the preapproval by the institutional research committee at CGMH. Informed written consent was provided for all blood donors by Medical Ethics and Human Clinical Trial Committee of our institution. We also obtained written informed consent from the parents or guardians of the participants under 18 years of age.

**Table 1:** Clinical and laboratory characteristics of SLE patients and controls.

Characteristics	Healthy controls (N = 13)	SLE patients (n = 26)
**Sex (Male/Female)**	5/8	3/23
**Age (Median, Range)**	29 (21–43)	34 (21–56)
**SLEDAI (Median, Range)**	N/A	6 (1–18)
**C3, Median (mg/dl, Range)**	N/A	70.8 (32.5–144.0)
**C4, Median (mg/dl, Range)**	N/A	11.5 (2.1–29.2)
**Anti-dsDNA (mg/dl, Range)**	N/A	215.9 (46.8–544.5)
**Malar Rash (%)**	N/A	15.4
**Malar Erythema (%)**	N/A	38.5
**Nephritis (%)**	N/A	46.2
**Arthritis (%)**	N/A	26.9
**Medication**		
**Prednisolone (%)**	N/A	92.3
**Azathioprine (%)**	N/A	50.0
**Mycophenolate Mofetil (%)**	N/A	7.7

### Preparation of iNKT cells

The preparation of iNKT cells were performed as previously described [14]. MNCs were obtained with Ficoll-Hypaque density gradient centrifugation and cultured in RPMI-1640 medium (GIBCO, Carlsbad, CA, USA) supplemented with 10% fetal bovine serum (FBS) (Biological Industries, Kibbutz Beit Haemek, Israel), penicillin (100 U/ml), and streptomycin (100 μg/ml, Biological Industries) in 6-well plates (1 x 10^6^ cells/well) in the presence of IL-15 (10 ng/ml) (PeproTech Inc., Rocky Hill, NJ, USA) with or without α-GalCer (100 ng/ml) (Funakoshi Co. Ltd. Tokyo. Japan) for 10 days, we dissolved α-GalCer in dimethyl sulfoxide (DMSO) at the concentration of 1 mg/ml. The supernatant was then discarded and the fresh medium with cytokines were added every 3 days. At the end of 10 days, cell count and viability were assessed by trypan blue exclusion. iNKT cell recovery ratio = The absolute viable Vβ11+/ Vα24^+^ cells count on day 10/ the absolute viable Vβ11+/ Vα24^+^ cells count on day 0.

### Surface marker analysis

On Day 10, the cells were harvested and re-suspended in phosphate-buffered saline (PBS), and stained with Vβ11^-^fluorescein isothiocyanate (FITC)/ Vα24^-^phycoerythrin (PE) (Beckman Coulter, Krefeld, Germany) and CD3^-^ FITC/ CD56^-^ PE (BD Pharmingen, San Diego, CA, USA) We also gated Vα24/ Vβ11^+^ cells for staining of FITC-anti-CD161 (BD pharmingen) respectively. After 20 minutes, the cells were washed with PBS twice and cultured with monoclonal antibodies at 4°C for 30 minutes. After that, the cells were washed twice again. We analyzed these cells with the FACS CANTOII flow cytometer (BD Biosciences, San Diego, CA, USA). Once the cells were enough, according to the forward scatter and side scatter profile, the data of 10,000 cells were collected on the basis of the gated lymphoid cell population.

### Intracellular cytokine staining

Phorbol myristate acetate (50 ng/ml) and ionomycin (1μg/ml, Sigma) were used to stimulate post-cultured cells. To avoid the secretion of the induced cytokine into the suspension, 2μM GolgiStop with monensin (BD pharmingen) was added. After cultured at 37°C for 4 hours, we harvested and labeled the cells by PE-anti-iNKT monoclonal antibody (Miltenyi Biotec, Bergisch Gladbach, Germany) at room temperature for 20 minutes. The cells then were washed with PBS once. The cells were fixed and permeabilized with 250μl solution at 4°C for 20 minutes, and then washed once with wash buffer. We stained the cells with FITC-anti-IFN-γ, FITC-anti-IL-4, FITC-anti-Granzyme B, and FITC-anti-Perforin (BD pharmingen). Later, the cells were washed once with PBS again, and we analyzed the samples by flow cytometry. Positive expression was shown as percentages.

### Detection of apoptosis

Post-cultured iNKT cells were identified with PE-anti-iNKT monoclonal antibody and incubated for 20 minutes at room temperature. The level of apoptosis was detected with the annexin-V (BD Pharminingen) and propidium iodide (PI; Sigma, St. Louis, MO, USA). To discriminate viable cells (annexin-V^-^/PI^-^) and early apoptotic cells (annexin-V^+^/PI^-^), the cells were stained with FITC annexin-V and the PI simultaneously. According to FSC/SSC characteristics, the PI^-^ population was gated, and the degree of apoptosis in viable MNCs was determined by flow cytometry.

### Flow-cytometric cytotoxicity assays

Cytotoxicity against K-562 cells was analyzed using flow cytometry as previously described [18]. The effector cells (E) and target cells (T) were mixed to obtain T:E ratio of 1:12,5 and 1:25, and incubated for 4 hours in complete medium. At the end of the incubation time, cells were stained for 20 min with FITC-CD45 antibodies. Propidium iodide (PI, 5ug/ml) were added to stained cells for DNA labeling of dead cells, and analyzed by flow cytometry. Cytotoxic Index of iNKT cells = (K562 cytotoxicity in the presence of α-GalCer—K562 cytotoxicity in the absence of α-GalCer) / K562 cytotoxicity in the absence of α-GalCer.

### Statistical analysis

To compare the data before and after a treatment, the Wilcoxon signed-rank test was used. Also, we compared the difference between the cells from normal individuals and SLE patients, applying the Mann-Whitney rank-sum test. The data are showed as mean ± standard error of mean. All the calculation was performed by SPSS 20.0 software. When the P value between two groups was less than 0.05, the difference was defined as significant.

## Results

### Deficient α-GalCer-induced expansion of iNKT cells and poor response to IL-15 in SLE patients

**Fig 1:**
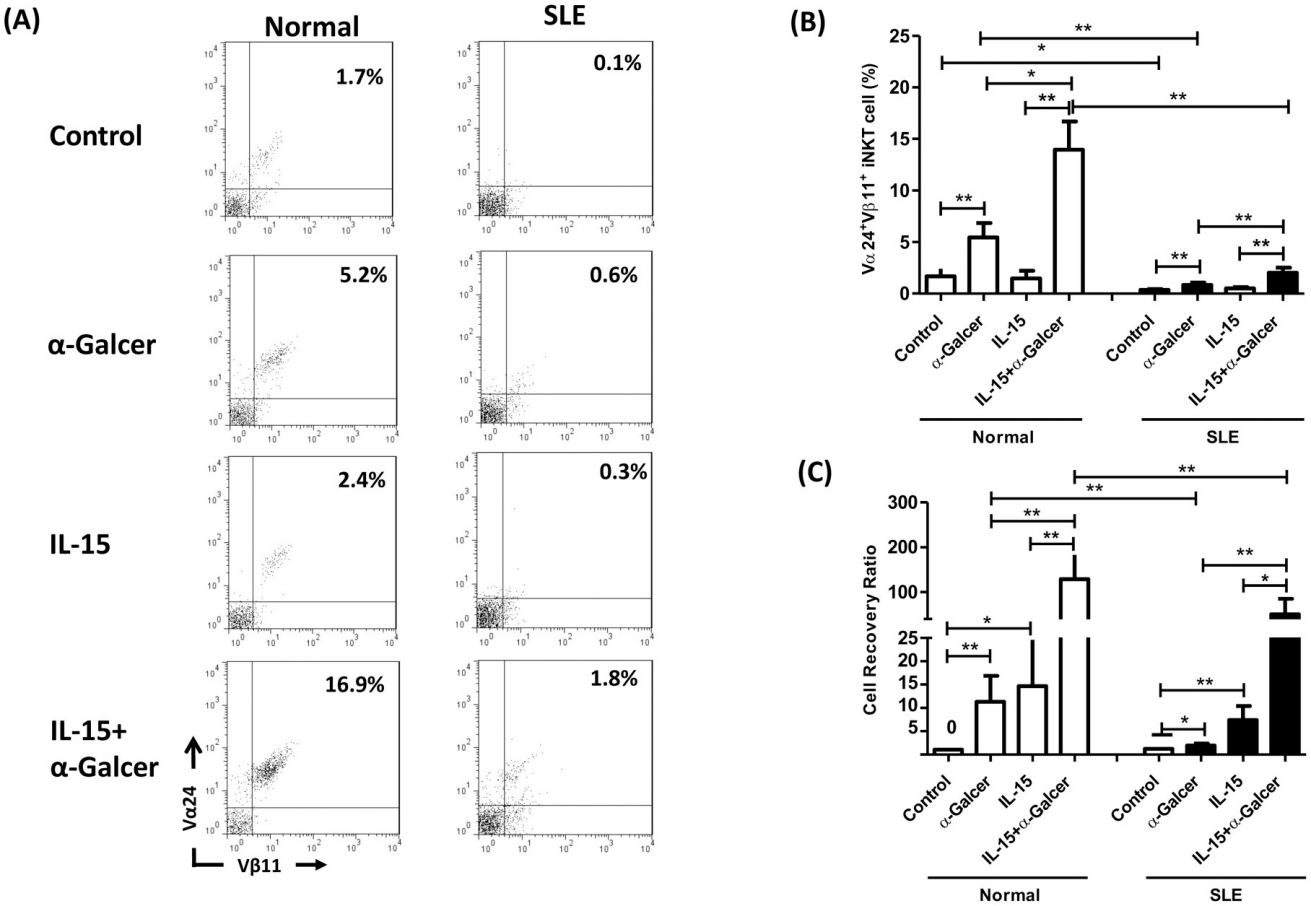
Deficient α-GalCer-induced expansion of iNKT cells and poor response to IL-15 in SLE patients. (A) A representative profile of dot-plot quadrant analysis for two-colored immunophenotyping of Vα24 and Vβ18 in SLE patients (SLE) and healthy controls (Normal). (B)The percentages of Vα24+/Vβ18+ iNKT cells (C) iNKT cell recovery ratio (see text) in each culture condition. MNCs were cultured in the presence or absence of IL-15 (10 ng/ml) with or without α-GalCer (100 ng/ml) for 10 days and then harvested for flow cytometric analysis. Healthy individuals, n = 13; SLE, n = 26; * P< 0.05, ** P <0.01.

Fig 1A showed the representative profile of dot-plot quadrant analysis on the effect of α-GalCer and IL-15 on the expansion of iNKT cells from normal controls and SLE patients. α-GalCer enhanced the percentages of Vα24^+^/Vβ11^+^ iNKT cells in controls (5.5±1.4% vs. 1.7±0.7%, p = 0.002) and SLE patients (0.8±0.2% vs. 0.4±0.1%, p = 0.001) as well, though the response of SLE patients was much lower (0.8±0.2% vs 5.5±1.4%, p<0.001) (Fig 1B). IL-15 alone had no effect on the percentages of Vα24^+^/Vβ11^+^ iNKT cells (see supplementary data). However, the combination of IL-15 and α-GalCer resulted in greater increase of the percentages of iNKT cells compared to α-GalCer alone in controls (14.0±2.7% vs 5.5±1.4%, p = 0.016) and SLE patients as well (2.0±0.5% vs 0.8±0.2%, p = 0.008). Again, the response of SLE was much poorer than controls (2.0±0.5% vs 14.0±2.7%, p<0.001).

As shown in Fig 1C, the iNKT cell recovery ratio in α-GalCer activated SLE MNC was decreased compared to that observed in controls (2.0±0.4 vs. 11.3±5.6, p = 0.002). In contrast to that observed with cell percentages, addition of IL-15 alone enhanced the iNKT cell recovery ratio in controls (14.7±9.9 vs. 1.1±0.2, p = 0.013) and SLE patients (7.4±3.0 vs. 1.2±0.4, p = 0.001) as well. Again, the combination of IL-15 and α-GalCer resulted in greater increase of iNKT cell recovery ratio compared to α-GalCer alone in controls (128.7±54.2 vs. 11.3±5.6, p = 0.002) and SLE patients (50.4±34.7 vs. 2.0±0.4, p<0.001) as well. The response of SLE was lower compared to corresponding controls (50.4±34.7 vs. 128.7±54.2, p = 0.007).

### Effect of IL-15 +α-GalCer stimulation on the percentages of CD3^+^/CD56^+^ NKT-like cells in SLE MNCs

The natural killer T-like (NKT-like) cells are CD3^+^ T cells coexpressing NK cell markers (CD56). We have previously shown that addition of α-GalCer did not further increase IL-15-treated CD3^+^/CD56^+^ NKT-like cells in healthy controls [14]. In the present study, we found that CD3^+^/CD56^+^ cells are slightly deficient in SLE patients as a whole compared to controls (1.6±0.3% vs 2.4±0.5%, p = 0.086), and IL-15 increased the percentages of CD3^+^/CD56^+^ NKT-like cells from SLE patients to a lesser degree compared to healthy controls (10.1±1.1% vs 18.6±2.5%, p = 0.006).

**Fig 2:**
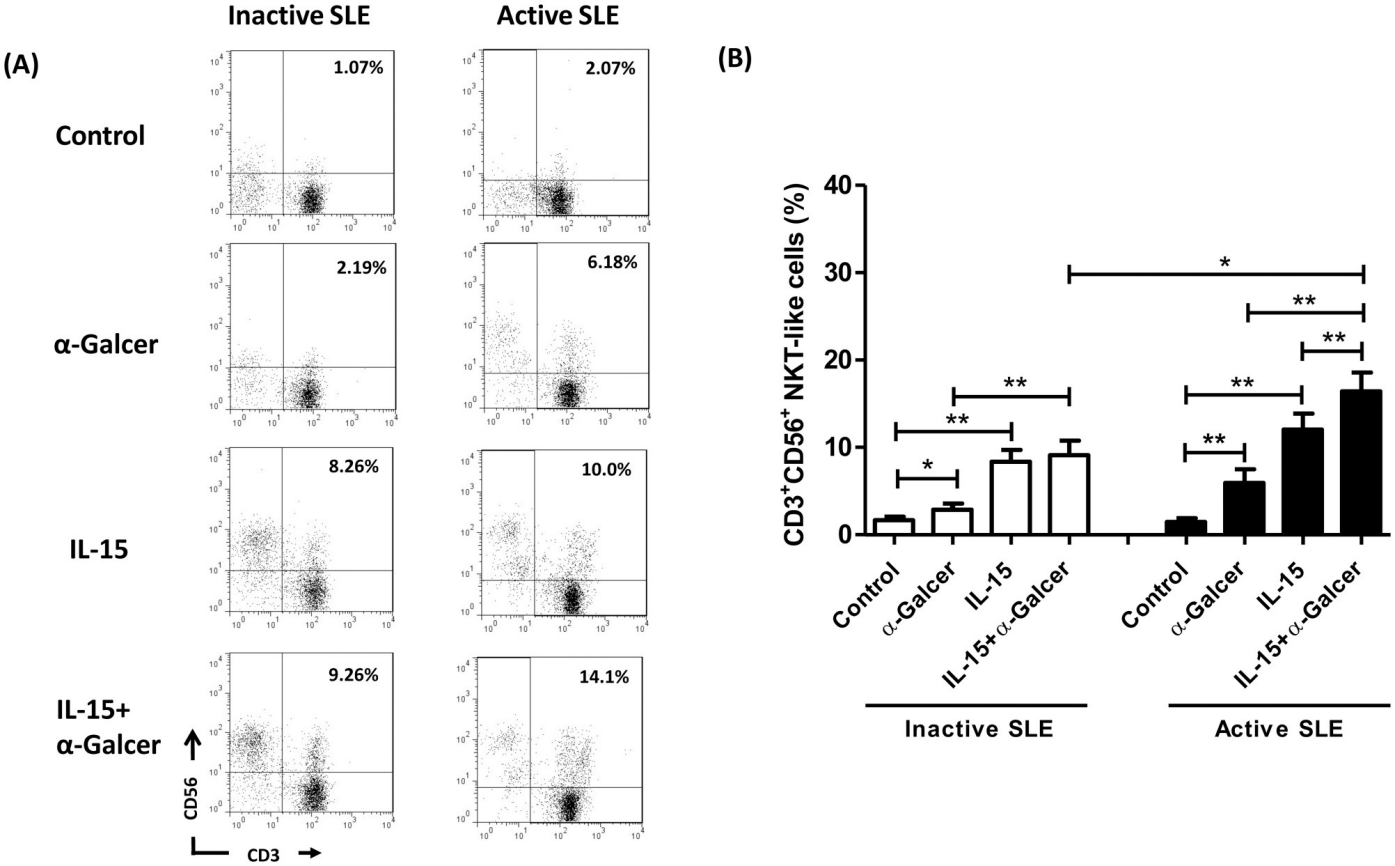
Effect of IL-15 +α-GalCer stimulation on the percentages of CD3^+^/CD56^+^ NKT-like cells in SLE MNCs. (A) A representative profile of dot-plot quadrant analysis for two-colored immunophenotyping of CD3 and CD56 according to disease activity of SLE patients (SLE). (B) The effect of IL-15 +α-GalCer stimulation on the percentages of CD3^+^/CD56^+^ NKT-like cells in SLE MNCs according to the disease activity. MNCs were cultured in the presence or absence of IL-15 (10 ng/ml) with or without α-GalCer (100 ng/ml) for 10 days and then harvested for flow cytometric analysis. Inactivate SLE, n = 14; active SLE, n = 12; *P< 0.05, **P <0.01.

Fig 2A shows a representative profile of the dot-plot quadrant analysis on the effect of IL-15 and α-GalCer on expansion of the NKT-like cells from SLE patients with active disease and inactive disease. For both groups, α-GalCer alone slightly increase the percentages of CD3^+^/CD56^+^ NKT-like cells in SLE MNCs. Addition of IL-15 alone expanded the percentages of CD3^+^/CD56^+^ NKT-like cells to a greater degree compared to α-GalCer alone. α-GalCer + IL-15 further increase the percentage of NKT-like cells compared to IL-15 alone in SLE patients with active disease (16.4±2.2% vs 12.1±1.8%, p = 0.025), which was not observed in inactive SLE disease(9.1±1.6% vs 8.4±1.3%, p = 0.413) (Fig 2B).

### Defective response to α-GalCer in SLE CD161^+^ iNKT cells restored by IL-15

**Fig 3:**
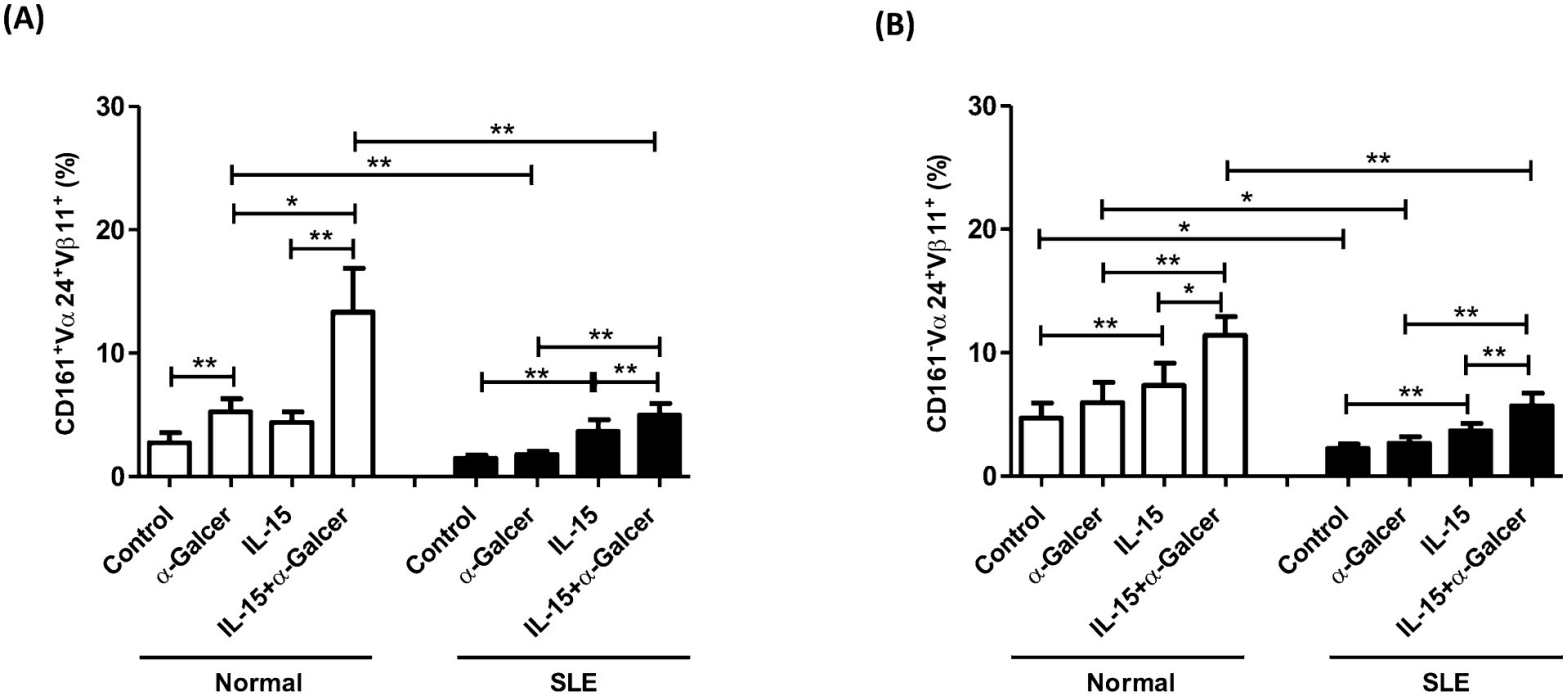
Defective response to α-GalCer in SLE CD161^+^ iNKT cells restored by IL-15. (A) The percentages of CD161^+^/ Vα24 ^+^ Vβ11^+^ iNKT cells (B) The percentages of CD161^-^/ Vα24 ^+^ Vβ11^+^ iNKT cells in SLE patients (SLE) and healthy controls (Normal). MNCs were cultured in the presence or absence of IL-15 (10 ng/ml) with or without α-GalCer (100 ng/ml) for 10 days and then harvested for flow cytometric analysis. Healthy individual, n = 11; SLE, n = 21; *P< 0.05, **P <0.01.

Human CD161^+^ iNKT cells are intrinsically endowed with the capacity to generate IL-17, a proinflammatory cytokine that may be involved in SLE pathogenesis. We next divided Vα24^+^/Vβ11^+^ iNKT cells into CD161^+^ and CD161^-^ subsets, and compared their differential response to IL-15 and α-GalCer (Fig 3). As shown in Fig 3A, α-GalCer enhanced the percentages of CD161^+^ iNKT subsets in controls (5.2±1.0% vs. 2.8±0.8%, p = 0.004) but not in SLE patients (1.8±0.3% vs. 1.5±0.3%, p = 0.304). IL-15 was superior to α-GalCer alone in increasing the percentages of CD161^+^ iNKT cells in controls (13.3±3.5% vs 5.2±1.0% p = 0.011) and SLE patients (5.0±1.0% vs 1.8±0.3%, p = 0.001). Thus, IL-15 enhanced the responsiveness of SLE CD161^+^ iNKT subsets to α-GalCer stimulation.

CD161^-^ iNKT subsets, in contrast to CD161^+^ iNKT subsets, did not respond to α-GalCer stimulation alone in controls (6.0±1.6% vs 4.7±1.2%, p = 0.059) and SLE patients(2.7±0.5% vs 2.3±0.4%, p = 0.158) (Fig 3B) Similar to that observed with CD161^+^ iNKT subsets, addition of IL-15 to α-GalCer cultures resulted in greater expansion of CD161^-^ iNKT subsets compared to α-GalCer alone in controls(11.4±1.5% vs 5.9±1.6%, p = 0.002) and SLE patients (5.7±1.0% vs 2.7±0.5%, p = 0.002).

### IL-15 decreased early apoptosis of α-GalCer-treated iNKT cells in SLE patients

**Fig 4:**
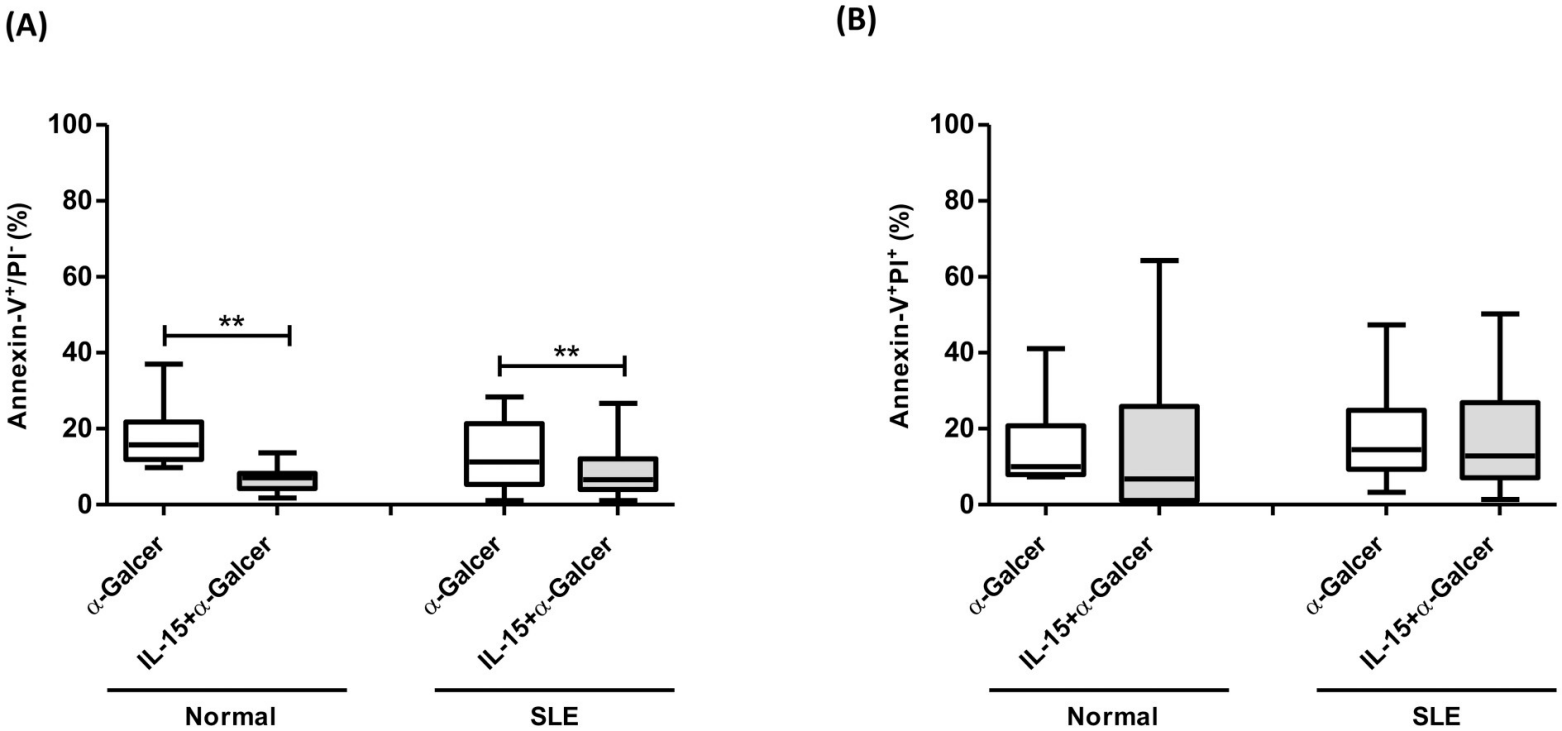
IL-15 decreased early apoptosis of α-GalCer-treated iNKT cells in SLE patients. The proportions of iNKT cells undergoing apoptosis in SLE patients (SLE) and healthy controls (Normal) under α-GalCer in the presence or absence of IL-15. (A) Annexin-V^+^/ PI^-^ early apoptotic cells (B) Annexin-V^+/^ PI^+^ late apoptotic cells. MNCs were cultured in the presence or absence of IL-15 (10 ng/ml) with or without α-GalCer (100 ng/ml) for 10 days and then harvested for flow cytometric analysis. Healthy individuals, n = 12; SLE, n = 26; *P< 0.05, **P <0.01.

Expanded iNKT cells were gated for Annexin-V/PI staining. Annexin-V^+^/PI^-^ and Annexin-V^+^/PI^+^ cells standed for early and late apoptosis, respectively. As shown in Fig 4A, the percentages of iNKT cells undergoing early and late apoptosis were similar in controls and SLE patients, respectively. IL-15, when added to α-GalCer cultures could significantly decrease the percentages of early apoptotic iNKT cells in both control (6.9±0.9% vs 18.1±2.2%, p = 0.002) and SLE patients (8.3±1.1% vs 13.1±1.7%, p = 0.009). IL-15, however, did not influence the percentages of late apoptotic iNKT cells (Fig 4B).

### IL-15 enhanced CD69, CD1d, and CD11a expression of α-GalCer-treated iNKT cells in SLE patients

**Fig 5:**
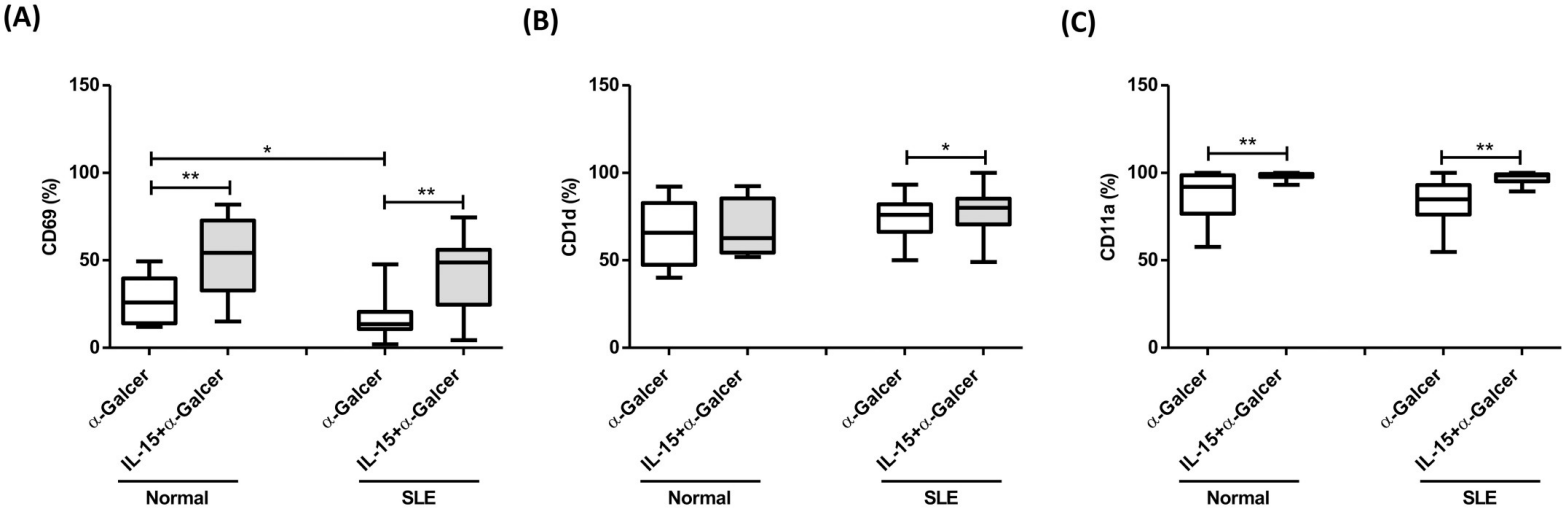
IL-15 enhanced CD69, CD1d, and CD11a expression of α-GalCer-treated iNKT cells in SLE patients. The production of(A) CD69, (B) CD1d and (C) CD11a in SLE patients (SLE) and healthy controls (Normal). Under α-GalCer in the presence or absence of IL-15. MNCs were cultured in the presence or absence of IL-15 (10 ng/ml) with or without α-GalCer (100 ng/ml) for 10 days and then harvested for flow cytometric analysis. Healthy individuals, n = 9; SLE, n = 21; *P< 0.05, **P <0.01.

As shown in Fig 5, CD69, CD1d, and CD11a expression of α-GalCer treated iNKT cells from SLE patients was comparable to that of controls, respectively. IL-15 enhanced CD69 expression of α-GalCer treated iNKT cells from SLE patients (41.4±4.6% vs. 17.2±2.7%, p = 0.001) as well as controls (51.1±6.5% vs. 26.6±3.8%, p = 0.005) (Fig 5A). IL-15 enhanced CD1d expression of α-GalCer treated iNKT cells from SLE patients (78.0±2.5% vs. 72.6±2.8%, p = 0.014) but not controls (68.3±5.2% vs. 65.5±6.0%, p = 0.374) (Fig 5B). IL-15 enhanced CD11a expression of α-GalCer treated iNKT cells from SLE patients (97.4±0.6% vs. 83.8±3.0%, p<0.001) as well as controls (98.4±0.5% vs. 86.5±4.1%, p = 0.004) (Fig 5C).

### IL-4, but not interferon-γ expression is deficient in iNKT cells from SLE patient

**Fig 6:**
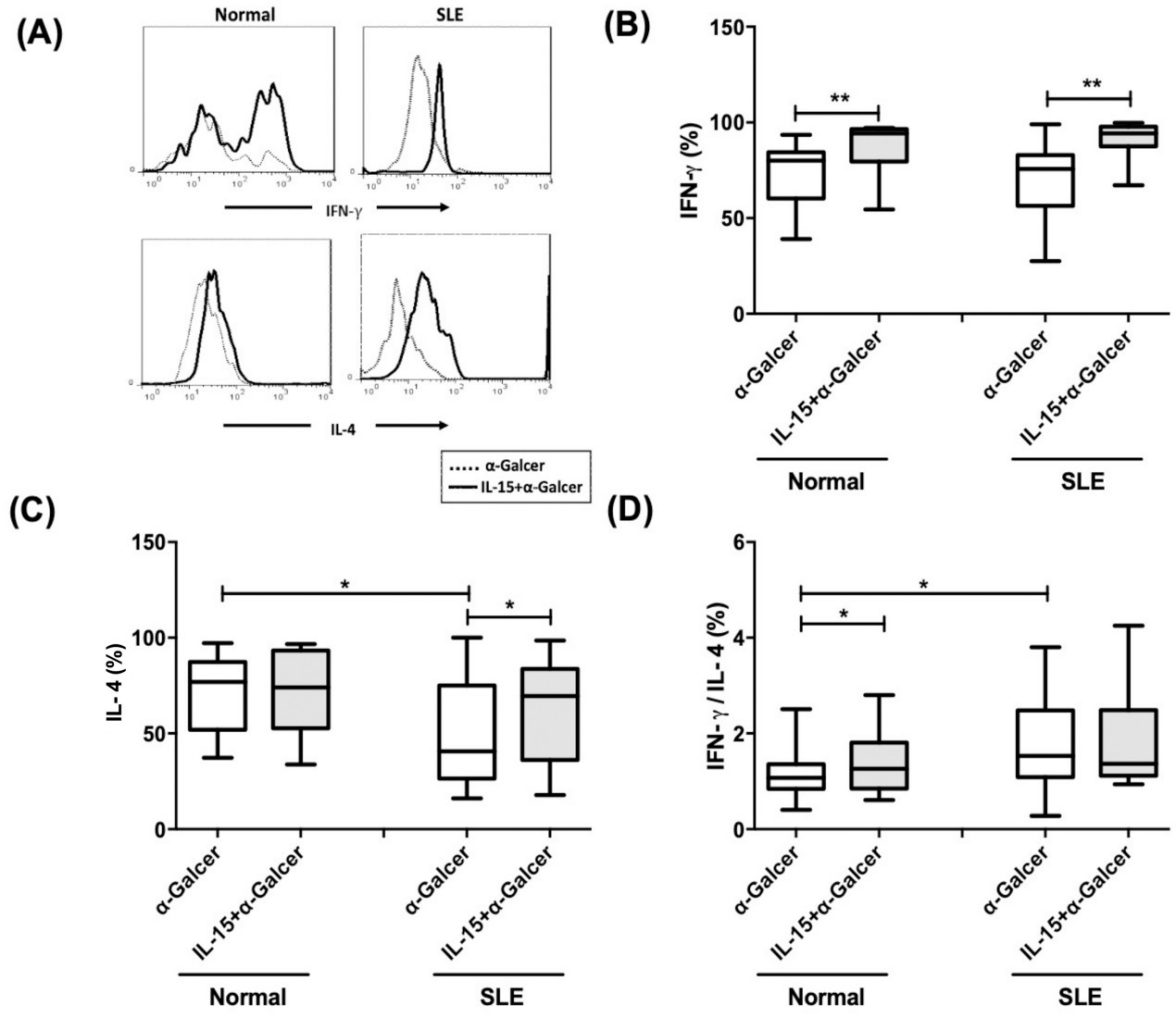
IL-4, but not interferon-γ expression is deficient in iNKT cells from SLE patient. (A) A representative histogram of flow cytometric analysis for intracellular staining of interferon-γ (IFN-γ) and interleukin-4(IL-4). Comparison was made between SLE patients and normal controls (Normal). The production of (B) IFN-γ, (C) IL-4, and (D) IFN-γ/ IL-4 ratio in SLE patients (SLE) and healthy controls (Normal). Under α-GalCer in the presence or absence of IL-15. MNCs were cultured in the presence or absence of IL-15 (10 ng/ml) with or without α-GalCer (100 ng/ml) for 10 days and then harvested for flow cytometric analysis. Healthy individuals, n = 13; SLE, n = 24; *P< 0.05, **P <0.01.

Fig 6A shows a representative profile of the dot-plot quadrant analysis for iNKT expansion and histograms of IFN-γ and IL-4 intracellular staining in control and SLE MNCs cultured with α-GalCer (100 ng/ml) without or with IL-15 (10 ng/ml) for 10 days. IFN-γ production of α-GalCer treated iNKT cells from SLE patients was comparable to that of controls (69.8±3.9% vs. 73.2±4.7%, p = 0.484). IL-15 enhanced IFN-γ production of α-GalCer treated iNKT cells from SLE patients (91.2±1.7% vs. 69.8±3.9%, p<0.001) as well as controls (86.5±3.8% vs. 73.2±4.7%, p = 0.003) (Fig 6B). In contrast, IL-4 production was significantly lower in SLE iNKT cells compared to controls (49.4±5.8% vs. 70.6±5.8%, p = 0.021). IL-15 increased IL-4 production of iNKT cells from SLE patients (62.7±5.7% vs. 49.4±5.8%, p = 0.04) but had little effect on controls (72.5±6.3% vs. 70.6±5.8%, p = 0.311) (Fig 6C).

We next analyzed the IFN-γ/IL-4 ratio of α-GalCer treated iNKT cells in each culture condtion (Fig 6D). The IFN-γ/IL-4 ratio was increased in α-GalCer treated iNKT cells on SLE patients compared to controls (1.8±0.2% vs. 1.2±0.2%, p = 0.019). No significant difference of IFN-γ/IL-4 ratio of α-GalCer treated iNKT cells in SLE patients was noted after addition of IL-15 (1.9±0.2% vs. 1.8±0.2%, p = 1.000). The IFN-γ/IL-4 ratio of α-GalCer treated iNKT cells of controls but not SLE patients, could be enhanced by IL-15, (1.4±0.2% vs. 1.2±0.2%, p = 0.033).

### α-GalCer-expanded iNKT cells from SLE patients had deficient perforin expression but comparable ability to enhance NK cytotoxicity

**Fig 7:**
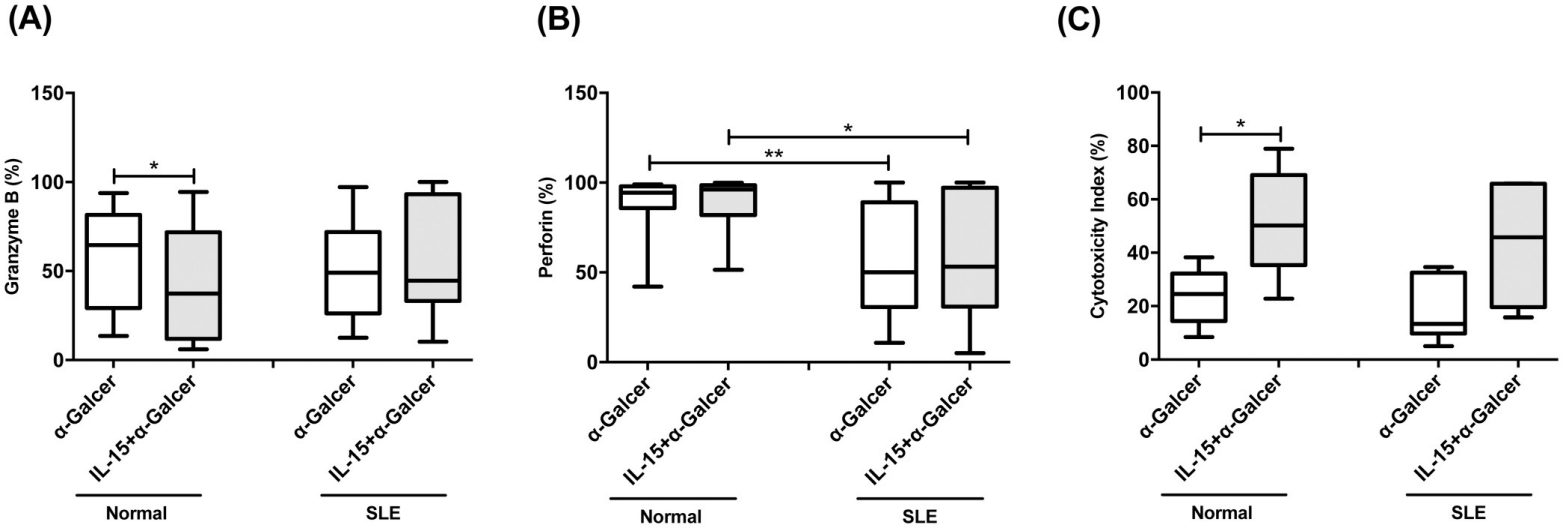
α-GalCer expanded iNKT cells from SLE patients had deficient perforin expression but comparable ability to enhance NK cytotoxicity. (A)The expression of granzyme B, (B) perforin and (C) Cytotoxic Index (see text) in SLE patients (SLE) and healthy controls (Normal) under α-GalCer in the presence or absence of IL-15. MNCs were cultured in the presence or absence of IL-15 (10 ng/ml) with or without α-GalCer (100 ng/ml) for 10 days and then harvested for flow cytometric analysis. Healthy individuals, n = 12; SLE, n = 25; *P< 0.05, **P <0.01.

We next compared the cytotoxic function of expanded iNKT cells from SLE patients and controls. Granzyme B expression of α-GalCer treated iNKT cells from SLE patients was comparable to that of controls (50.6±5.5% vs 56.6±9.3% p = 0.584). Addition of IL-15 had no effect on granzyme B expression of α-GalCer treated iNKT cells from SLE patients (55.7±6.2% vs, 50.6±5.5% p = 0.264) but decreased that of controls (44.1±9.9% vs 56.6±9.3% p = 0.013) (Fig 7A).

In contrast, perforin expression was significantly lower in SLE iNKT cells compared to controls (56.6±6.0%vs 87.1±5.0% p = 0.003). IL-15 had no effect on perforin expression of iNKT cells from SLE patients (59.0±6.5% vs 56.6±6.0% P = 0.558) and controls (88.8±4.6% vs87.1±5.0%, p = 0.239), respectively. (Fig 7B).

To evaluate the enhancing effect of α-GalCer on NK cytotoxic function, we perform flow cytometric analysis using K562 as target cells, which is sensitive to NK cell but not iNKT cells. The iNKT dependent cytotoxic index was calculated as:

(%cytolysisinthepresenceofα-GalCer−%lysisintheabsenceofα-GalCer)/%cytolysisinthepresenceofα-GalCerX100.

The cytotoxic index, therefore, reflected the ability of iNKT cell to enhance autologous NK cytotoxicity. As shown in Fig 7C, the cytotoxic index of SLE iNKT cells was comparable to controls. (18.8±3.8% vs 23.2±3.6%, p = 0.29). However, they were not response to IL-15 stimulation (42.9±8.1% vs 18.8±3.8%, p = 0.128) as control did (51.0±6.7% vs 23.2±3.6% p = 0.017).

### CD161, granzyme B and perforin expression of α-GalCer + IL-15 treated iNKT cells correlate with markers of SLE disease activity

**Fig 8:**
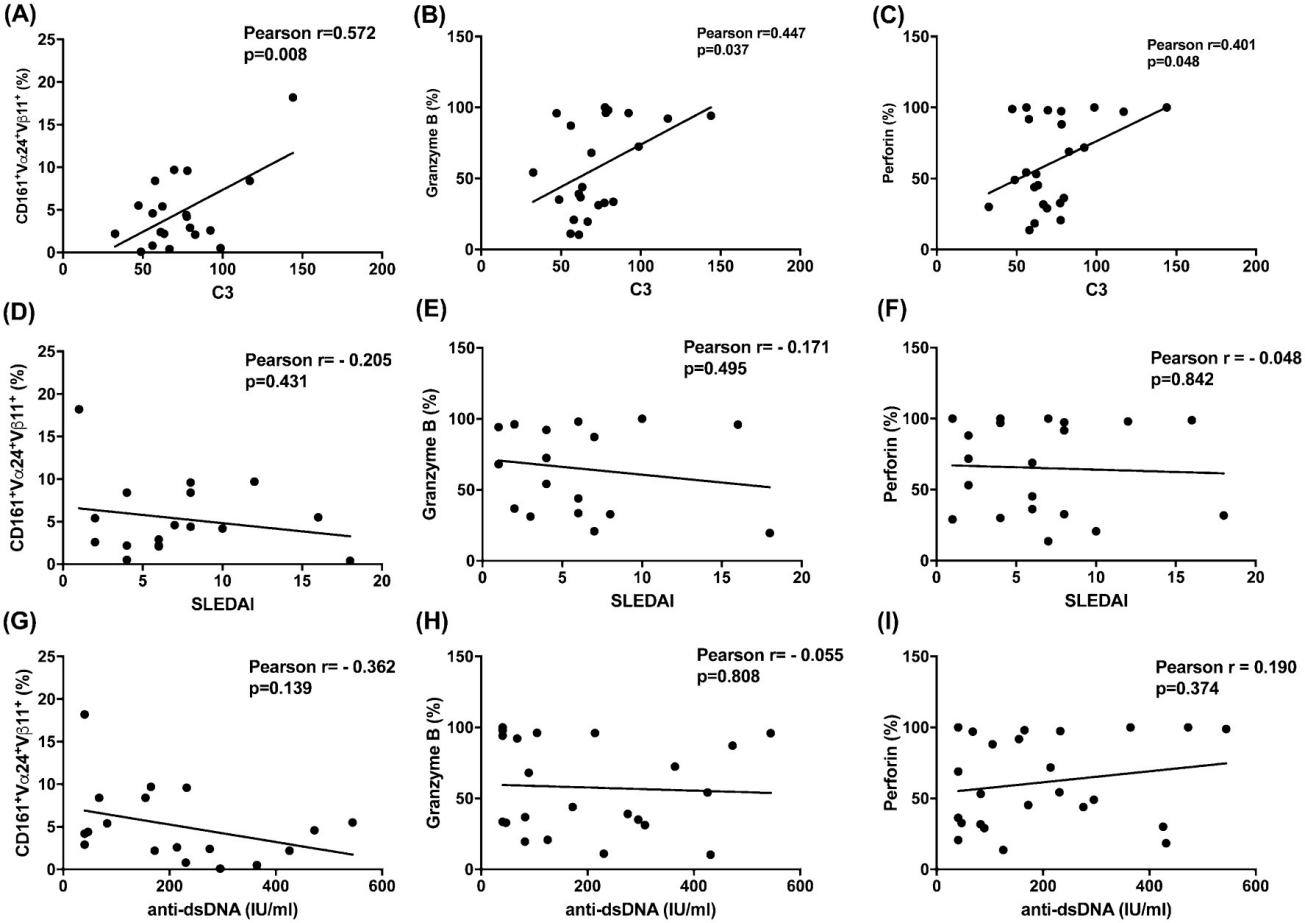
CD161, granzyme B and perforin expression of α-GalCer + IL-15 treated iNKT cells correlate with markers of SLE disease activity. Correlation of (A) the percentages of CD161^**+**^ cells (B) Granzyme B and (C) perforin expression of α-GalCer +IL-15 expanded iNKT cells with complement C3 level, correlation of (D) the percentages of CD161^**+**^ cells, (E) Granzyme B, and (F) perforin expression of α-GalCer + IL-15 expanded iNKT cells with SLEDAI and correlation of (G) the percentages of CD161^**+**^ cells (H) Granzyme B and (I) perforin with anti-dsDNA. MNCs were cultured in the presence or absence of IL-15 (10 ng/ml) with or without α-GalCer (100 ng/ml) for 10 days and then harvested for flow cytometric analysis, n = 26.

Finally, we examined the correlation between the various parameters of expanded iNKT cells from SLE patients and their laboratory workup indicating disease activity, and SLEDAI as well. As shown in Fig 8, the expression of CD161 on α-GalCer + IL-15 expanded iNKT cell cells positively correlated with C3 (r = 0.572, *p* = 0.008) (Fig 8A). The C3 was also positively correlated to granzyme B (r = 0.447, *p* = 0.037) and perforin (r = 0.401, *p* = 0.048) expression onα-GalCer + IL-15 expanded iNKT cells, respectively (Fig 8B and 8C). The expression of CD161 on α-GalCer + IL-15 expanded iNKT cells, perforin and granzyme B was not correlated to anti-dsDNA level and SLEDAI in SLE pateints (Fig 8D–8I).

## Discussion

The role of iNKT cell in SLE pathogenesis is not fully understood as studies in SLE animal model have yielded conflicting results. Yang et al showed that repeated α-GalCer injection alleviated inflammatory dermatitis in MRL-lpr/lpr mice [19] and brief exposure to α-GalCer confers protection against lupus development [20]. However, Zeng et al showed that activation of iNKT in NZB/W mice exacerbated lupus [21].

Previous studies found decreased number of iNKT cells in both NZB/NZW F1 lupus mice [22] and humans with SLE [23]. Reduction of Vα14 iNKT cell numbers in the MRL/lpr of lupus models was correlated with the progression of autoimmunity, and the injection of Vα14 TCR transgenic iNKT cells could alleviate the onset of clinical symptoms of lupus in MRL/lpr mice [24].

The limitation of this study is that we did not recruit newly diagnosed, treatment naïve SLE patient, so that the effect of medication on iNKT cells from SLE patients cannot be ruled out. Immunosuppressive medication was correlated to the number of log-transformed absolute iNKT cells [8]. However, lower iNKT cell number was noted in SLE patients without drug use compared to healthy controls. Wither *et al* found that there was no correlation between medication use and the proportion of NKT cells in SLE patients [25].

We found that iNKT cells in SLE patients were deficient in number and showed poor response to α-GalCer stimulation, in agreement with previous studies [23, 26]. We and others have previous shown the ability of IL-15 to regulate the phenotype and function of NK and NK T-like -cells in SLE patients [15, 27, 28]. Although IL-15 alone did not increase iNKT cell percentage in MNCs, it expanded the absolute counts of iNKT cells, and thus increased cell recovery. IL-15 also further enhance α-Galcer-induced iNKT proliferation in SLE, but to a lesser extent compared to controls.

CD3^+^/CD56^+^ NKT-like cells are important innate immune effectors capable of producing large amount of cytokines and exhibit cytotoxicity against tumor cells [1, 29]. The response of NKT-like cells to IL-15+ α-GalCer in SLE patients with inactive disease was similar to that observed in healthy controls [14]. IL-15+ α-GalCer was not superior to IL-15 alone in increasing NKT-like cell percentage. However, addition of IL-15 further increased the percentages of α-Galcer-treated NKT-like cells in SLE patients with active SLE disease, suggesting NKT-like cells from SLE patients with active disease are more readily activated by IL-15.

In normal controls, the CD161^+^ iNKT subsets, but not CD161^-^ iNKT subsets responded to α-GalCer simulation. However, CD161^+^ iNKT subsets in SLE patient did not response to α-GalCer simulation. CD161, also refer to as C-type lectin domain family 5 member B, is an iNKT cell marker gradually expressed during differentiation from fetus [30]. Previous studies have shown the deficient expression of CD161 on NK and T cells from SLE patients [31]. Our finding demonstrated an intrinsic defect of CD161^+^ iNKT subsets in SLE patients.

HIV infection substantially decreases iNKT cell numbers and functions possibly through induction of apoptosis [32]. Patients with active *M*. *tuberculosis* infection also showed reduction in iNKT cell numbers due to increased iNKT cell apoptosis [33]. We found that α-Galcer-treated iNKT cells in SLE patients were not particularly prone to apoptosis compared to controls. IL-15 decreased the percentages of early apoptotic iNKT cells in SLE patients as well as controls. In this regard, IL-15 may be beneficial in SLE patients as they are generally considered immunocompromised. The anti-apoptotic effect of IL-15 was not seen in late apoptotic cells. IL-15 has been shown to possess anti-apoptotic properties for CD8 T cells and NK cells [34].

iNKT cells express surface markers such as CD25, and CD69, which are characteristic of activated and memory T cells [35]. We showed that CD69 expression of α-Galcer-treated iNKT cells was deficient compared to controls, but could be enhanced by IL-15. Delivering α-GalCer with strong co-stimulation such as recombinant CD1d molecules can potently activate iNKT cells [36]. CD1d expression on α-Galcer-treated iNKT cells from SLE patients were comparable to controls and could be further enhanced by IL-15. CD11a is an important integrin that regulates iNKT cell adhesion and thereby cytotoxicity against tumor or virus-infected cells [37]. IL-15 enhanced the CD11a expression of α-Galcer-treated iNKT cells from SLE patients.

Activated iNKT cells simultaneously secrete IFN-γ and IL-4 [38]. Impaired ability to proliferate and produce IFN-γ in response to α-GalCer stimulation was observed in patients with tuberculosis [33]. We found that IL-4, but not IFN-γ, production of α-GalCer expanded iNKT cells from SLE patients was decreased compared to controls. IL-15 enhanced IFN-γ production but had no effect on IL-4, indicating a Th1-biased response. Guimarães et al showed that plasma IFN-γ was increased while IL-4 decreased in in SLE patients [39]. IFN-γ secreted by activated iNKT cells suppress pathogenic T cells and inhibit Th17 response in certain autoimmune diseases [40, 41].

Activated iNKT cells can also kill target cells through their expression of perforin/granzyme [42]. Structural changes of autoantigens by granzyme B and perforin may be involved in the pathogenesis of SLE [43]. Blanco et al. demonstrate that greater proportions of perforin- and/or granzyme B-positive lymphocytes are correlated to the higher SLE Disease Activity Index [44]. We found, however, a decreased perforin expression of expanded SLE iNKT cells, suggesting an impaired cytotoxic function. iNKT cells can exert their cytotoxic function by granule exocytosis and kill CD1d expressing tumor directly [45]. They may also enhance autologous NK cell cytotoxic function [14]. K562 cytotoxicity assay revealed that the ability of SLE iNKT cells to enhance NK killing against K562 cells (iNKT resistant) was comparable to controls. Finally, the percentages of CD161^+^ iNKT cells, perforin and granzyme B expression of expanded iNKT cells correlated with complement C3 levels, thus may confer protection against lupus flare.

Taken together, these results demonstrate numeric deficiency and aberrant function of iNKT cells under the influence of IL-15 in SLE patients. The beneficial effect of IL-15 to help expand iNKT cells may be compromised by using IL-15 antagonist as immunotherapy in SLE patients.

## Supporting information

S1 Fig (PDF)

S2 Fig (PDF)

S3 Fig (PDF)

S4 Fig (PDF)

S5 Fig (PDF)

S6 Fig (PDF)

S7 Fig (PDF)

S8 Fig (PDF)
