# Supplementary material for: Expansion of invariant natural killer T cells from systemic lupus erythematosus patients by alpha-Galactosylceramide and IL-15
Source: PLoS One. 2021 Dec 22;16(12):e0261727. doi: 10.1371/journal.pone.0261727 (PMC8694473; doi:10.1371/journal.pone.0261727)
Supplement: S1 Fig — (PDF) [file pone.0261727.s001.pdf]

Fig1(B)

Normal	va24/vb11			
	media	KRN	IL-15	IL-15+KRN
	0.2	0.2	0.3	32
	0.7	2.6	0.8	16.3
	0.1	0.7	1	9.8
	0.7	2.5	0.7	1.9
	0.2	1.2	0.5	19.4
	0.1	2.1	2.8	10.9
	0.8	2.5	0.9	3
	0.3	10	0.3	4.3
	1.6	2.9	0.9	9.7
	0.7	9.5	0.5	33.6
	7.8	12.7	10.1	11.5
	7.3	14.6	0.3	16.2
	1.3	9.4	0.1	13.1

SLE	va24/vb11			
	media	KRN	IL-15	IL-15+KRN
	0.7	5.9	0.7	0.5
	0.1	0.1	0	0.3
	0.1	0.3	0	0.1
	0.6	1.1	0	1
	0.6	1.1	0	1
	1.2	0.9	0.1	0.3
	0.3	0.3	0.5	0.5
	1.3	1.1	1.5	1.5
	0.1	1.4	0.4	5.2
	0.1	0	0.2	0.2
	0.1	0.1	0.2	6.6
	0.2	2.3	0.2	6.4
	0.2	0.4	1.1	2.1
	0.2	0.1	0.2	10.3
	0.2	0.7	0.1	0.1
	0	0.5	0.1	2.2
	0	0.3	0	2.6
	0	0.3	0	0.1
	0.2	0.3	2.5	2
	0.2	0.3	1.2	1.1
	0.3	0.6	0.3	0.9
	0.4	0.6	0.5	1.4
	1	0.9	1.3	2.7
	0.3	0.8	0.5	1.1
	0.7	0.9	1.2	0.6
	0.5	0.6	0.5	2.2

Fig1(C)

Normal	va24/vb11			
	media	KRN	IL-15	IL-15+KRN
	0.9	4.0	1.4	462.2
	0.1	0.7	0.8	22.0
	1.2	5.5	34.2	278.5
	0.7	2.3	3.9	8.7
	0.7	5.0	6.3	183.0
	2.9	75.0	130.0	591.7
	0.5	6.4	1.7	17.7
	1.7	24.4	4.3	35.0
	0.8	1.5	0.5	5.5
	1.3	10.6	1.5	45.7
	2.0	4.9	6.2	11.2
	0.9	4.3	0.1	4.3
	0.2	2.6	0.1	8.1

SLE	va24/vb11			
	media	KRN	IL-15	IL-15+KRN
	1.7	2.0	44.0	13.9
	2.9	4.4	67.1	61.5
	0.3	2.0	1.5	0.7
	0.2	0.4	1.2	2.9
	0.6	1.0	0.0	5.9
	2.2	2.3	8.4	24.6
	0.5	0.4	1.8	2.5
	1.9	5.7	0.0	6.7
	0.4	1.0	3.1	5.8
	2.1	5.2	11.4	2.7
	0.2	0.3	0.0	1.8
	0.1	0.4	0.0	0.7
	0.5	0.4	0.1	0.3
	0.7	0.4	2.7	3.3
	3.2	2.7	3.9	3.9
	0.3	4.0	2.7	34.7
	0.6	0.0	3.1	3.1
	0.6	0.6	2.6	85.2
	0.9	1.1	4.1	17.9
	0.5	5.8	3.0	96.9
	1.9	3.9	14.0	26.7
	9.4	4.7	17.6	908.8
	0.7	2.5	0.8	0.8
	0.0	0.0	0.0	0.0
	0.0	0.0	0.0	0.0
	0.0	0.0	0.0	0.0
