# Supplementary material for: Expansion of invariant natural killer T cells from systemic lupus erythematosus patients by alpha-Galactosylceramide and IL-15
Source: PLoS One. 2021 Dec 22;16(12):e0261727. doi: 10.1371/journal.pone.0261727 (PMC8694473; doi:10.1371/journal.pone.0261727)
Supplement: S2 Fig — (PDF) [file pone.0261727.s002.pdf]

Fig2(B)

Inactive SLE

CD3+CD56+ NKT-like cell			
media	KRN	IL-15	IL-15+KRN
0.6	0.7	2.8	4.1
4.3	6.9	19.3	26.3
2	3.9	11.5	11.6
1	5.6	14.2	11.7
2.1	2.4	7.8	8.6
3.9	8	8.9	9
1.9	2.2	10.9	10.1
0.9	1.1	9	8.7
4	5.9	8.4	13.2
0.7	1.6	7.9	7.1
0.4	0.1	0.7	0.8
0.4	0.4	1.6	1.9
0.6	0.6	4.6	5
0.9	0.9	9.8	9.6

Active SLE

CD3+CD56+ NKT-like cell			
media	KRN	IL-15	IL-15+KRN
0.3	0.6	8.4	8.1
2.4	4.7	17.8	27.5
0.4	5.6	4.8	6.6
1.1	2.6	14.6	19.6
0.6	15.9	14.1	26
0.5	3.3	6.8	8.3
0.5	6.5	6.6	22.5
0.9	2.6	9.4	12.2
5.4	15.3	15.1	22.6
2.8	11.9	27.7	19.2
1	1.3	11.1	15.8
1.8	1.2	8.3	8.6
