# Supplementary material for: Expansion of invariant natural killer T cells from systemic lupus erythematosus patients by alpha-Galactosylceramide and IL-15
Source: PLoS One. 2021 Dec 22;16(12):e0261727. doi: 10.1371/journal.pone.0261727 (PMC8694473; doi:10.1371/journal.pone.0261727)
Supplement: S3 Fig — (PDF) [file pone.0261727.s003.pdf]

Fig3(A)

Normal

CD161+Va24Vb11			
media	KRN	IL-15	IL-15+KRN
0.5	0.4	2.2	16.1
0.8	3.5	2.7	8.8
1	6.3	2.5	10.6
2	2.3	1.3	1.4
2.3	4.7	5.4	18
0.4	5.5	8.5	13.6
2.5	3.3	2.1	4.2
1.5	2.6	3.6	5.8
4.7	8.9	5.3	6.5
5	7	10.5	17.3
9.4	13.2	4.1	44.5

SLE

CD161+Va24Vb11			
media	KRN	IL-15	IL-15+KRN
0.7	1.1	2	2.2
0.6	2.4	0.3	0.8
3	3.4	0.6	2.4
0.4	0.2	0.1	0.1
0.2	0.7	0.3	0.4
0.7	1.3	1.8	4.4
0.8	0.7	2.2	2.1
1.4	0.6	0.9	2.2
3	3.1	4	5.4
0.7	1	3	2.9
0.5	1.2	1.8	2.6
0.7	1.2	2.5	4.2
0.2	0.2	0.4	0.5
1	2.4	9	8.4
1.8	3.8	14.1	18.2
1.9	1	3.4	4.6
1.9	0.9	3.7	5.5
4.6	2.2	15.8	9.7
2.6	3.1	6.5	8.4
1.1	5	1.8	9.6
3.3	2.1	2.6	9.9

Fig3(B)

Normal

CD161-Va24Vb11			
media	KRN	IL-15	IL-15+KRN
2.8	4.2	4.9	21.6
2.4	3	4.5	8.7
2.4	2.9	5.8	11.9
4.2	7.8	5.1	12.3
9.8	9.4	17.9	14.5
4.2	3.9	5.1	6.3
17.5	23.8	24.2	21.9
4.2	5.2	5.1	10.8
1.4	5.5	9.3	10.9
5.6	5.7	5.4	8
3.5	4.7	5.2	7
1.2	1.1	1.6	3.1
2	0.4	1.5	11.5

SLE

CD161-Va24Vb11			
media	KRN	IL-15	IL-15+KRN
1.6	0.8	5.8	16.5
5	10	6.8	8.1
5.8	6.1	4.9	4.8
2.5	1.2	0.8	3.9
2.3	3.1	2.9	3.2
3.7	5.2	6.2	12.7
1.8	1.4	4.2	3.6
3	2	3.1	9
3.5	4.8	6.3	10
3.5	4.8	6.3	10
2.9	3.2	7.6	12.6
3.5	2.9	9.6	9.3
3.4	3.8	3.7	5
0.5	0.9	1.1	1
0.7	1.3	1.3	1.9
0.9	1	1.2	1.3
0.7	0.6	1.2	1.6
0.9	0.8	2.3	0.1
0.9	0.8	2	2.1
0.1	0.9	0.1	2.3
0.1	0.6	0.1	0.9
