# Supplementary material for: Expansion of invariant natural killer T cells from systemic lupus erythematosus patients by alpha-Galactosylceramide and IL-15
Source: PLoS One. 2021 Dec 22;16(12):e0261727. doi: 10.1371/journal.pone.0261727 (PMC8694473; doi:10.1371/journal.pone.0261727)
Supplement: S4 Fig — (PDF) [file pone.0261727.s004.pdf]

Fig4(A)

Normal

Annexin-V+PI-	
KRN	IL-15+KRN
10.9	8.2
15.3	7.5
16.3	6.1
9.8	9.2
15.2	13.7
22	5.9
37	3.5
25.6	7.9
18.2	8.3
15.2	6.8
10.3	3.8
21	1.8

SLE

Annexin-V+PI-	
KRN	IL-15+KRN
19.2	9.3
12	4
27.3	5.4
3.7	1.1
4.5	6.7
6.1	7.9
24	5
11.9	12.4
28.1	11.5
9	6.5
20.5	9.7
10.7	4.5
6.8	3.7
24.4	26.7
12.6	6.3
1.1	1.1
3.6	4
5.7	2.4
5.8	7.6
4.2	14.6
3.9	15.2
18.2	3.6
13	15.8
10.5	6.2
28.4	13.2
25.5	12

Fig4(B)

Normal

Annexin-V+/PI+	
KRN	IL-15+KRN
14.5	8.1
22.8	31.3
31.4	39.2
7.8	2.2
9.8	0.8
41.1	64.3
7.4	0.8
9.3	5.5
8.5	9.5
7.4	7.6
14.8	5.9
10.3	0.6

SLE

Annexin-V+/PI+	
KRN	IL-15+KRN
47.4	50.3
16.9	9.5
15.2	5.4
7.4	7.7
22.7	9.5
20.4	23.6
20	10.8
14.8	9.8
9.4	12.4
3.3	1.4
30.5	23
9.3	9.4
9.3	20.2
28.8	36.8
23.6	16.7
5.9	5
5.8	5
11.7	2.4
9.6	4.5
14.1	13.6
14.3	48.1
44.3	38.2
36	42
9.6	13.3
12.1	19.7
29.4	39.9
