# Supplementary material for: Expansion of invariant natural killer T cells from systemic lupus erythematosus patients by alpha-Galactosylceramide and IL-15
Source: PLoS One. 2021 Dec 22;16(12):e0261727. doi: 10.1371/journal.pone.0261727 (PMC8694473; doi:10.1371/journal.pone.0261727)
Supplement: S5 Fig — (PDF) [file pone.0261727.s005.pdf]

Fig5(A)

Normal

CD69	
KRN	IL-15+KRN
45.5	33.1
12.4	35.9
18.9	54.3
27	66.5
33.9	80.2
49.4	81.9
47.3	79.1
27.2	66.2
25.9	34
12.4	21
15.8	15.1
18	32.4
12.1	64.5

SLE

CD69	
KRN	IL-15+KRN
12.1	41.5
12.5	56
15.5	54.5
6.1	50
7	48.8
47.7	56.5
13.2	59
7.3	4.4
13.9	37.2
20.6	19.9
20.3	63.2
25	9.62
13.5	39
11.7	24.7
24	31.7
10.8	51.2
44.3	55.8
19.4	74.5
2.1	9.3

Fig5(B)

Normal

CD1d	
KRN	IL-15+KRN
63.6	51.9
40	52.2
48.3	56.6
82.6	82.3
46.5	61.9
65.8	66.4
67.2	62.7
92.2	88.5
82.9	92.4

SLE

CD1d	
KRN	IL-15+KRN
66.7	73.5
65.9	80
50	49.1
50.1	61.9
50	66.7
70	83.3
73.6	72
85.7	100
76.1	78.6
76.9	67.5
71.2	76.1
86.7	69.2
79.7	83.9
84.4	86.6
77.3	80.2
53.7	74.1
76.3	80.5
74.7	86.6
77.5	88.8
85.7	95.3
93.3	83.5

Fig5(C)

Normal

CD11a	
KRN	IL-15+KRN
58.8	98.8
78.4	99.3
75	97.8
100	100
93.8	99.5
99.2	99
100	100
98.2	99.1
89.4	99.4
92	98.8
93.8	97.2
88.1	93.2
57.7	97.6

SLE

CD11a	
KRN	IL-15+KRN
90.8	93.4
84.4	99.5
73	95.2
75	94.4
77.8	89.4
99.5	98.6
84	97.6
84.4	98.7
89.3	96.9
94.3	98.7
87.5	94.7
76.2	98.9
93.1	100
100	99.6
89.9	99.1
84.9	99.2
54.9	98.3
98.9	99.8
54.8	98.2
