# Supplementary material for: Expansion of invariant natural killer T cells from systemic lupus erythematosus patients by alpha-Galactosylceramide and IL-15
Source: PLoS One. 2021 Dec 22;16(12):e0261727. doi: 10.1371/journal.pone.0261727 (PMC8694473; doi:10.1371/journal.pone.0261727)
Supplement: S6 Fig — (PDF) [file pone.0261727.s006.pdf]

Fig6(B)

Normal

IFN-r	
KRN	IL-15+KRN
48.2	54.5
92.1	96.7
39	81
65.4	65.6
57.6	78
78.3	96.7
82.8	81
93.6	94.8
84.1	96.3
80	94.3
82.8	97.2
84.8	95.2
63	93.7

SLE

IFN-r	
KRN	IL-15+KRN
71.5	88.8
60.8	94.8
55	80.5
75.9	81.4
43.8	94
60.5	75.7
75.7	98.1
97.1	96
27.6	87.1
79.6	98.3
82.9	98.2
81.1	93.4
82.8	97.2
90.7	94.6
88.7	92.3
75.7	97.3
83.8	97.9
75.6	98.9
99.1	99.8
66.9	95.9
34.3	92
72	90
50.4	67.2
43.8	78.9

Fig6(C)

Normal

IL-4	
KRN	IL-15+KRN
86	90
88.7	95
97.2	96.7
94.6	92.5
48	52.8
37.9	41.9
76.9	94.1
37.3	33.8
78.1	52.6
75.1	52.8
83.7	92.6
55.9	73.3
58.7	74.1

SLE

IL-4	
KRN	IL-15+KRN
49.5	43.9
16	25.5
26	19.9
28.2	22.9
25	29.8
55.6	17.8
100	97.3
75	51.7
99.7	77.1
40.7	94.4
82.5	74.2
53	83.7
26.4	69.5
24.9	80.8
93	98.3
30.5	87.2
58.7	78.2
49.3	80.8
93.7	98.5
30.4	67.7
27.4	58.4
27.8	36.2
22.2	49.1

Fig6(D)

Normal

IFN-r/IL-4	
KRN	IL-15+KRN
0.6	0.6
1.0	1.0
0.4	0.8
0.7	0.7
1.2	1.5
2.1	2.3
1.1	0.9
2.5	2.8
1.1	1.8
1.1	1.8
1.0	1.0
1.5	1.3
1.1	1.3

SLE

IFN-r/IL-4	
KRN	IL-15+KRN
1.4	2.0
3.8	3.7
2.1	4.0
2.7	3.6
1.8	3.2
1.1	4.3
0.8	1.0
1.3	1.9
0.3	1.1
2.0	1.0
1.0	1.3
1.5	1.1
3.1	1.4
3.6	1.2
1.0	0.9
2.5	1.1
1.4	1.3
1.5	1.2
1.1	1.0
2.2	1.4
1.3	1.6
2.6	2.5
2.3	1.4
