# Supplementary material for: Expansion of invariant natural killer T cells from systemic lupus erythematosus patients by alpha-Galactosylceramide and IL-15
Source: PLoS One. 2021 Dec 22;16(12):e0261727. doi: 10.1371/journal.pone.0261727 (PMC8694473; doi:10.1371/journal.pone.0261727)
Supplement: S7 Fig — (PDF) [file pone.0261727.s007.pdf]

Fig7(A)

Normal

Granzyme B	
KRN	IL-15+KRN
33.9	34.6
90.3	79
93.8	94.4
13.5	6.1
70	62.9
59	34.7
78.6	40
32.1	6.1
74.8	69.4
20.2	13.8

SLE

Granzyme B	
KRN	IL-15+KRN
61	34
61.7	44.6
36.6	45.8
32.7	43.9
12.5	11.1
63.6	39
32	35
27.9	19.6
16.7	10.3
15.7	20.8
18.6	68
53.8	31.2
41.4	32.8
17	33.5
40.7	54.2
24.5	36.8
71.7	98.1
94.9	96
72.2	100
55.6	72.4
97.1	92.2
49	94.2
91.3	87.2
78.4	96.2
97.2	95.9

Fig7(B)

Normal

Perforin	
KRN	IL-15+KRN
42	51.4
62.8	78.3
95.9	92.9
92.9	94.7
86.6	62.7
98.1	96.8
97.3	98.8
89.5	97
85.6	95.6
99.1	99.9
96.2	99
98.9	97.9

SLE

Perforin	
KRN	IL-15+KRN
57.9	45.3
55.4	54.3
42.7	43.9
40.5	49.1
23.3	31.8
50	18.4
30	13.7
45.3	29.1
16.7	5.1
29.4	32.7
35.6	68.9
42.9	30
68.4	53.2
13.7	36.3
31.3	71.8
10.8	20.7
76.5	100
96.1	97
92.9	100
100	100
95.9	88.2
95.7	98.9
81.8	98
85.1	91.8
97.9	97.4

Fig7(C)

Normal

Cytotoxicity index	
KRN	IL-15+KRN
24.0	79.0
25.2	22.8
25.0	32.8
38.3	43.1
14.0	56.1
8.5	49.5
15.7	73.5
34.5	51.0

SLE

Cytotoxicity index	
KRN	IL-15+KRN
11.6	15.8
11.9	
13.4	65.9
19.3	57.7
5.0	
7.9	65.9
32.8	19.6
34.6	29.8
32.3	45.8
