# Supplementary material for: Expansion of invariant natural killer T cells from systemic lupus erythematosus patients by alpha-Galactosylceramide and IL-15
Source: PLoS One. 2021 Dec 22;16(12):e0261727. doi: 10.1371/journal.pone.0261727 (PMC8694473; doi:10.1371/journal.pone.0261727)
Supplement: S8 Fig — (PDF) [file pone.0261727.s008.pdf]

Fig8(A)

C3	CD161
63.4	2.2
56	0.8
61.2	2.4
48.8	0.1
66.7	0.4
61.3	
58	
69	
73.5	
77.3	4.4
82.8	2.1
32.6	2.2
62.3	5.4
79.6	2.9
92.4	2.6
77.6	4.2
98.7	0.5
117	8.4
144	18.2
56.2	4.6
78.2	
47.3	5.5
69.6	9.7
57.7	8.4
77.9	9.6

Fig8(B)

C3	Granzyme B
63.4	43.9
56	11.1
61.2	39
48.8	35
66.7	19.6
61.3	10.3
58	20.8
69	68
73.5	31.2
77.3	32.8
82.8	33.5
32.6	54.2
62.3	36.8
79.6	98.1
92.4	96
77.6	100
98.7	72.4
117	92.2
144	94.2
56.2	87.2
78.2	96.2
47.3	95.9
69.6	
57.7	
77.9	
32.5	

C3	Perforin
63.4	45.3
56	54.3
61.2	43.9
48.8	49.1
66.7	31.8
61.3	18.4
58	13.7
69	29.1
73.5	
77.3	32.7
82.8	68.9
32.6	30
62.3	53.2
79.6	36.3
92.4	71.8
77.6	20.7
98.7	100
117	97
144	100
56.2	100
78.2	88.2
47.3	98.9
69.6	98
57.7	91.8
77.9	97.4
32.5	

Fig8(D)

SLEDAI	CD161
6	2.2
	0.8
	2.4
	0.1
18	0.4
7	
1	
3	
8	4.4
6	2.1
4	2.2
2	5.4
6	2.9
2	2.6
10	4.2
4	0.5
4	8.4
1	18.2
7	4.6
2	
16	5.5
12	9.7
8	8.4
8	9.6
8	

Fig8(E)

SLEDAI	Granzyme B
6	43.9
	11.1
	39
	35
18	19.6
	10.3
7	20.8
1	68
3	31.2
8	32.8
6	33.5
4	54.2
2	36.8
6	98.1
2	96
10	100
4	72.4
4	92.2
1	94.2
7	87.2
2	96.2
16	95.9
12	
8	
8	
8	

Fig8(F)

SLEDAI	Perforin
6	45.3
	54.3
	43.9
	49.1
18	31.8
	18.4
7	13.7
1	29.1
3	
8	32.7
6	68.9
4	30
2	53.2
6	36.3
2	71.8
10	20.7
4	100
4	97
1	100
7	100
2	88.2
16	98.9
12	98
8	91.8
8	97.4
8	

Fig8(G)

anti-dsDNA	CD161
172.1	2.2
230.7	0.8
275.7	2.4
295.4	0.1
431.4	
125	
89.5	
308.1	
46.8	4.4
425.8	2.2
82.7	5.4
40.5	2.9
214.1	2.6
40.5	4.2
364.4	0.5
67.9	8.4
40.5	18.2
472.9	4.6
105.1	
544.5	5.5
165.3	9.7
154.8	8.4
232.6	9.6
290.1	

Fig8(H)

anti-dsDNA	Granzyme B
172.1	43.9
230.7	11.1
275.7	39
295.4	35
82.6	19.6
431.4	10.3
125	20.8
89.5	68
308.1	31.2
46.8	32.8
40.5	33.5
425.8	54.2
82.7	36.8
40.5	98.1
214.1	96
40.5	100
364.4	72.4
67.9	92.2
40.5	94.2
472.9	87.2
105.1	96.2
544.5	95.9
165.3	
154.8	
232.6	
290.1	

Fig8(I)

anti-dsDNA	Perforin
172.1	45.3
230.7	54.3
275.7	43.9
295.4	49.1
82.6	31.8
431.4	18.4
125	13.7
89.5	29.1
308.1	
46.8	32.7
40.5	68.9
425.8	30
82.7	53.2
40.5	36.3
214.1	71.8
40.5	20.7
364.4	100
67.9	97
40.5	100
472.9	100
105.1	88.2
544.5	98.9
165.3	98
154.8	91.8
232.6	97.4
290.1	
